# Computational identification of microRNAs in Anatid herpesvirus 1 genome

**DOI:** 10.1186/1743-422X-9-93

**Published:** 2012-05-14

**Authors:** Jun Xiang, Anchun Cheng, Mingshu Wang, Shunchuan Zhang, Dekang Zhu, Renyong Jia, Shun Chen, Yi Zhou, Xiaoyu Wang, Xiaoyue Chen

**Affiliations:** 1 Institute of Preventive Veterinary Medicine, Sichuan Agricultural University, Wenjiang, Chengdu city, Sichuan, 611130, People’s Republic of China; 2 Avian Disease Research Center, College of Veterinary Medicine of Sichuan Agricultural University, 46 Xinkang Road, Ya’an, Sichuan, 625014, People’s Republic of China; 3 Key Laboratory of Animal Disease and Human Health of Sichuan Province, Sichuan Agricultural University, Wenjiang, Chengdu city, Sichuan, 611130, People’s Republic of China

**Keywords:** Anatid herpesvirus 1, microRNA, Conservation, Computational analyses

## Abstract

**Background:**

MicroRNAs (miRNAs) are a group of short (~22 nt) noncoding RNAs that specifically regulate gene expression at the post-transcriptional level. miRNA precursors (pre-miRNAs), which are imperfect stem loop structures of ~70 nt, are processed into mature miRNAs by cellular RNases III. To date, thousands of miRNAs have been identified in different organisms. Several viruses have been reported to encode miRNAs.

**Findings:**

Here, we extended the analysis of miRNA-encoding potential to the Anatid herpesvirus 1 (AHV-1). Using computational approaches, we found that AHV-1 putatively encodes 12 mature miRNAs. We then compared the 12 mature miRNAs candidates with the all known miRNAs of the herpesvirus family. Interestingly, the “seed sequences” (nt 2 to 8) of 2 miRNAs were predicted to have the high conservation in position and/or sequence with the 2 miRNAs of Marek’s disease virus type 1 (MDV-1). Additionally, we searched the targets from viral mRNAs.

**Conclusions:**

Using computational approaches, we found that AHV-1 putatively encodes 12 mature miRNAs and 2 miRNAs have the high conservation with the 2 miRNAs of MDV-1. The result suggested that AHV-1 and MDV-1 should have closed evolutionary relation, which provides a valuable evidence of classification of AHV-1. Additionally, seven viral gene targets were found, which suggested that AHV-1 miRNAs could affect its own gene expression.

## Findings

MicroRNAs (miRNAs) are noncoding small RNA molecules with important regulatory functions in expression of target genes [1,2]. The miRNAs are about 19 to 25 nucleotides (nt) long. They are firstly transcribed as long primary miRNAs, which are processed into 60–70 nt miRNA precursor (pre-miRNA) by nuclear RNase III Drosha [3]. Pre-miRNA is then exported to the cytoplasm by the export factor Exportin 5 and further cleaved into ∼ 22 nt duplexes [4]. Mature miRNAs regulate protein-coding gene expression via the RNA silencing machinery, typically by forming imperfect duplexes with target messenger RNAs (mRNAs).

To date, thousands of miRNAs have been identified in different organisms [5]. The discovery of miRNAs encoded by DNA viruses suggests that viruses have evolved to exploit RNA silencing for regulation of the expression of their own genes, host genes, or both [6]. Most viral miRNAs (vmiRNAs) have been identified by cDNA cloning of small RNAs from virus-infected cells [7-10], whereas others have been identified following computational prediction and hybridization analysis [10-12]. Experimental screening of vmiRNAs via high-throughput sequencing of large numbers of cDNA clones from infected cells is technically challenging, time consuming and could be incomplete, given that viral gene expression can have highly constrained tissue-, time-, and replication state-specific patterns [12]. Of the vmiRNAs identified so far, most are encoded by viruses in the herpesvirus family, containing α [13-16], β [17], γ subfamily [8,9,18]. AHV-1, an unassigned virus in the family Herpesviridae, can induce duck viral enteritis in waterfowl of the family Anatidae. To query whether this strategy is also employed by AHV-1 or not, we have analyzed putative miRNA-encoding capacity of AHV-1.

**Figure 1:**
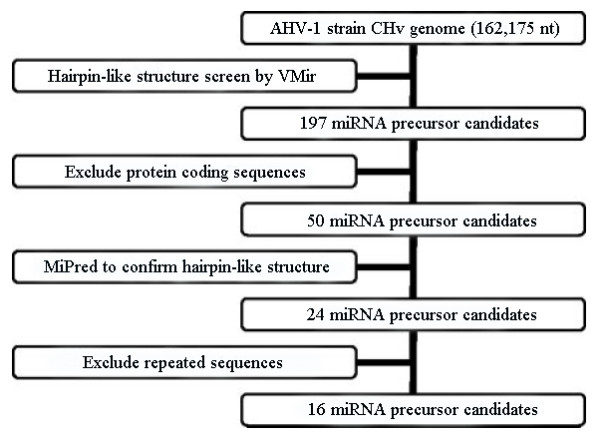
Flowchart of the AHV-1 miRNA prediction procedure.

**Figure 2:**
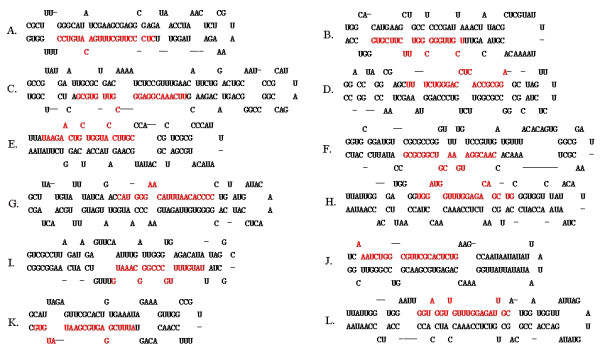
**Secondary structure predictions of AHV-1 pre-miRNAs.** The putative mature miRNAs sequence were shown in red. **A-L** in order named AHV-1-pre-miR-1 to AHV-1-pre-miR-12.

The AHV-1 miRNA prediction was performed using the complete genome sequence of AHV-1 strain CHv (JQ647509) [19]. The genome size is 162,175 nt. Figure 1 shows a flowchart of the computational prediction process. Briefly, the viral genome was scanned for hairpin-structured miRNA precursors using VMir Analyzer program [20,21]. 197 sequences with potential hairpin-like structures were extracted as candidate miRNA precursors. Then candidates within or antisense to protein-coding regions were removed according to the NCBI genome annotations. 50 precursors were further identified using MiPred program (http://www.bioinf.seu.edu.cn/miRNA/) and the sequences with lower minimum free energy (equivalent or below −25 kcal.mol^-1^) were remained, subsequently, the remained 24 real pre-microRNA sequences were conducted BLASTn searching against itself to remove repeated sequences. Finally, 16 sequences were selected as miRNA precursors candidates. At the last step, the mature sequences were predicted by Bayes-SVM-MiRNA web server v1.0 (http://wotan.wistar.upenn. edu/BayesSVMmiRNAfind/). After that, 12 mature sequences were predicted with 21nt in length (Table 1) and the secondary structure of pre-miRNAs were shown in Figure 2.

**Table 1:** Sequences and genomic positions of putative AHV-1 miRNAs

**No.**	**Predicted mature miRNA** **sequence (5’ to 3’)**	**Position,** **orientation**	**Location**
1	CUCCCUUGCUUUGACAUGUCC	26325-26345, -	Within intergenic region between *UL44* and *UL45*
2	UCGUUGGGCGGUUUCUUCGUG	72302-72322, -	Within intergenic region between *UL26* and *UL27*
3	UUCAAACGGAGGCGUUGUGCG	72512-72532, +	Within intergenic region between *UL26* and *UL27*
4	UUUCUGGGACCUCACCGCGGA	79262-79282, +	Within intergenic region between *UL22* and *UL23*
5	UAAGAACUGCUGGUACCUUGC	112559-112579, +	Within intergenic region between *UL4* and *UL5*
6	CAACGGAUGAACGUCGGCGCG	112720-112740, -	Within intergenic region between *UL4* and *UL5*
7	CAUGGGAACAUUUAACACCCC	123128-123148, +	Within intergenic region between *ICP22* and *LORF2*
8	UGGAUGGUUUGGAGACAGCUG	125173-125193, +	Within intergenic region between *ICP4* and *ICP22*
9	UAUGUUUUGCCCGGGCAAAUG	132907-132927, +	Within intergenic region between *US1* and *ICP4*
10	AAAUCUGGCGUUCGCACUCUG	134522-134542, -	Within intergenic region between *US1* and *ICP4*
11	AUUUCGGAGUGCGAAUAUGUG	134586-134606, -	Within intergenic region between *US1* and *ICP4*
12	GGUAGGUUGUUUGGAGAUUGC	160321-160341, +	Within unique short terminal repeat region

In order to investigate whether the AHV-1 miRNAs predicted are conserved in other herpesviruses, each of the 12 putative mature AHV-1 miRNA candidates was compared with the all known miRNAs of the herpesvirus family independently in database (http://www.sanger.ac.uk/Software/Rfam/mirna/). Interestingly, the “seed sequences” (nt 2 to 8) of 2 miRNAs were predicted to have the high conservation with the 2 miRNAs of MDV-1 (Table 2). The genomic positions of the 2 miRNAs encoded by MDV-1 are proximal to the latencyassociated transcript region. The vmiRNAs have generally been reported to lack sequence conservation across different viral species [10], with the exception of the primate polyomaviruses [11]. But among closed viral species, they could showed conservation in position and/or sequence. Jurak *et al* identified 16 and 17 miRNAs expressed by herpes simplex viruses 1 and 2 (HSV-1 and −2), respectively. The genomic positions of most miRNAs encoded by these two viruses are within or proximal to the latency associated transcript region. Nine miRNAs are conserved in position and/or sequence, particularly in the seed region, between these two viruses [16]. Additional, Waidner *et al* reported the genome locations, but not microRNA sequences, are conserved among four avian herpesviruses, infectious laryngotracheitis virus (ILTV) and herpesvirus of turkeys (HVT), as well as Marek's disease viruses (MDV-1 and MDV-2). Most are clustered in the repeats flanking the unique long region (I/TRL), except in ILTV which lacks these repeats [14]. So the result suggested that AHV-1 and MDV-1 should have closed evolutionary relation, which provides a valuable evidence of classification of AHV-1. Meanwhile, the prevalence of microRNAs in the genomic repeat regions suggests that the latent infection in herpesviruses could be relevant to function of microRNA.

**Table 2:** miRNA homologs expressed by AHV-1 and MDV-1

**Name**	**miRNA sequence**^***a***^	**No. of identical nt/total** **in seed region (nt positions 2–8)**
AHV-1-pre-miR-7	-C**AUGGGAA**CAUU-UAACACCCC G**CAUGGAA**ACGUCCUGGGAAA- - * * * * * †* * * † * † * † † *	5/7
MDV-1-miR-M13		
AHV-1-pre-miR-9	U**AUGUUUU**GCCCGGGCAAAUG-U**CUGUUGU**UCCGUAGUGUUCUC * * * * * * * * † * † † †	5/7
MDV-1-miR-M6-5p		

^*a*^ Sequences of orthologous miRNAs expressed by AHV-1 and MDV-1 aligned using ClustalW2. The seed sequences are shown in boldface. Identical nucleotides and nucleotides with conserved target binding potential are indicated with * and †, respectively.

What is the function of the vmiRNA? In order to know whether the vmiRNA could modulate its own genes expression, we checked the 3’UTR of viral mRNAs that could perfectly complement with the “seed sequence” of vmiRNA. AHV-1-pre-miR-4 was predicted to target UL29 gene (DNA replication-recombination; binds single-stranded DNA) and US5 gene (unknown function). AHV-1-pre-miR-7 was predicted to target UL16 gene (capsid maturase). AHV-1-pre-miR-9 was predicted to target UL15B gene (DNA cleavage-encapsidation), UL45 (tegument/envelope protein), and US1 gene (immediate-early and late transrepressor protein). AHV-1-pre-miR-12 was predicted to target UL45 gene and US7 gene (cell-cell spread). However, none of gene targets were found for the other vmiRNAs. Additionally, we wonder whether the putative vmiRNA could be used by AHV-1 to modulate host cell genes expression profiles. But so far genome of duck is in the process of being annotated and there is not available 3’UTR database of duck genes, so prediction can not be carried on.

Here, we introduced a concept that the AHV-1 genome could reasonably encode candidate pre-miRNAs. Studies are in progress to experimentally identify the putative vmiRNAs during AHV-1 infection.

## Competing interests

The authors declare that they have no competing interests.

## Authors’ contributions

JX carried out most of the data collection, data analysis and drafted the manuscript. ACC, MSW, SCZ, DKZ, RYJ, SC, YZ, XYW and XYC helped draft the manuscript. All authors read and approved the final manuscript.
